# Odours count: human olfactory ecology appears to be helpful in the improvement of the sense of smell

**DOI:** 10.1038/s41598-021-96334-3

**Published:** 2021-08-19

**Authors:** Anna Oleszkiewicz, Lena Heyne, Beata Sienkiewicz-Oleszkiewicz, Mandy Cuevas, Antje Haehner, Thomas Hummel

**Affiliations:** 1 Smell and Taste Clinic, Department of Otorhinolaryngology, TU Dresden, Dresden, Germany; 2 Institute of Psychology, University of Wroclaw, Wrocław, Poland; 3 Department of Clinical Pharmacology, Faculty of Pharmacy, Wroclaw Medical University, Wrocław, Poland

**Keywords:** Olfactory system, Environmental impact, Psychology and behaviour

## Abstract

Odours modify human behaviour. Research in this field develops rapidly, providing more and more exciting discoveries. In this context, our daily odorous environment has been surprisingly poorly explored. The aim of our study was to quantify olfactory perception and preliminarily identify factors affecting the frequency of odorous experiences. We were also interested in knowing whether human olfactory ecology relates with olfactory performance. In this study, patients with olfactory deficits (n = 62) and healthy controls (n = 97) had their olfactory threshold and odour identification abilities measured before and after a two-week intervention comprising counting of conscious perception of odours naturally occurring in the environment. In both groups, we observed enhanced olfactory performance after the intervention suggesting that (1) the conscious focus on odours may change its perception, and that (2) social and physical environment can effectively stimulate the human olfactory system, presumably supporting the improvement of olfactory sensitivity.

## Introduction

People rarely reflect on the extent to which their actions and decisions may be driven by smells. Each day, we come across numerous odours that elicit certain memories^1,2^ and enhance certain behaviours^3–5^. The sense of smell is an important proxy regulating human interaction with the environment. Food odours elicit cerebral activity in brain reward circuits^6^ and increase food intake, especially the smell of high-energy dense foods^7,8^. The smell of gas or spoiled food elicits avoidance reactions^9^. Losing the ability to perceive odours inflates the risk of environmental hazards such as consuming spoiled food^10^ or unaware exposure to potentially toxic chemical agents^3,11,12^. Psychological costs of not being able to smell include compromised romantic and sexual activity^13,14^ and enhanced depressive and anxiety symptoms^15^.

Olfactory perception reflects personal experience with odours^16–19^. Olfactory experience is shaped in the course of odour-related activities in childhood and adulthood^20^. The way human–environment interactions are regulated by olfactory perception largely depends on the hedonic valence of odours (pleasant–unpleasant). Conscious perception of odours emerges because they are particularly good or bad and people either want to actively enjoy the smell or locate its source and move away from it^21^. Pleasant odours, associated with happiness and likely to occur in urban spaces (e.g. “bee wax”, “summer air”) have been found to evoke weaker autonomic response than unpleasant odours (e.g. "vomit", "burnt") that have connotation with disgust^22,23^.

Several psychometric tools have been designed to quantify the influence of odours on various daily life domains. The “Odour Awareness Scale” measures the tendency to notice, pay attention and attach importance to the olfactory sensations and using them to guide individual choices, actions and attitudes^24^. The “Individual Significance of Olfaction Scale” reflects emotions, memories and impressions related to odours (Association scale), the degree to which olfaction is used on a daily basis (Application scale), and the conclusions drawn from olfactory experience (Consequence scale)^25^. The "Odours in Everyday Life" questionnaire measures the role of odours in the assessment of the environment, everyday life practices, sexuality and social relationships^26^. The “Children’s Olfactory Behaviour in Everyday Life” (COBEL) has been designed to measure children’s engagement in olfactory-related behaviours^27^, likely to shape future olfactory perception. However, all these questionnaires neglect quantifying odorous perceptions in daily life but focus on their role in regulating human–environment interactions. Furthermore, these scales are based on self-reported measures, thus they are prone to recall bias^28^. As a consequence, an explorative account on the frequency of conscious olfactory experiences is missing.

Regular, structured exposure to odours for approximately 3 months, called olfactory training (OT), is used in clinical settings to enhance olfactory performance and rehabilitate the olfactory system^29–33^. Shorter and more ecologically valid, immersive exposure to a set of 72 predefined odours has also been shown to enhance olfactory function in subjects with olfactory deficits, suggesting that conscious perception of odours can improve olfactory performance in those individuals who exhibit poor olfactory performance at baseline^34^. This logic suggests that ambient, environmental odours may have beneficial effects for olfactory performance, however little is known about the quantity and quality of everyday olfactory perceptions and their effect on olfactory performance. In a study Mahmut et al.^35^ asked subjects to count their olfactory perceptions for two days prior to mindfulness meditation intervention and over two days post meditation intervention. Although mindful meditation did not increase odour identification abilities, or individual significance of olfaction, or the number of olfactory perceptions counted after the intervention, subjects declared that they noticed odors more often after mindful meditation. Importantly, the study by Mahmut et al. offers insight into the types of odours recorded by the participants. Almost half of the 2770 olfactory perceptions were associated with places (e.g. “room”, “hallway”), 22% were associated with urban places (e.g. “work”, “cinema”) and 21% were associated with food (e.g. “breakfast”, “cooking”, “coffee”), despite the direct instruction not to count food-related odours what highlights the role of retronasal olfaction in perception of daily odours. Other categories concerned personal hygiene, nature, humans and animals and objects^35^.

So far, the role of olfactory perceptions in regulating human behaviour has been demonstrated, but human olfactory ecology has been poorly described. Exploration of human olfactory ecology is important for our understanding where and how people come across everyday odours and in what circumstances these olfactory perceptions are conscious. Exploration of human olfactory ecology may therefore deepen our knowledge about the link between human olfactory ecology and odour-driven behaviour. To this end, we designed a study where we asked the participants to count conscious odour perceptions that they spontaneously experience in their environment. We invited patients with olfactory dysfunction and healthy controls to count their odour perceptions for two weeks and report basic information about the environment they were embedded in over this period, including the frequency of interpersonal contacts per day and differentiation of the environment to indoor, outdoor and mixed.

## Results

### Sample demographics

A total of 159 participants completed the study. The sample was balanced in terms of sex, χ^2^(1) = 1.46, *p* = 0.23. Patients were significantly older than the control subjects, *t*(157) =  − 4.4, *p* < 0.001, therefore age was controlled in the subsequent analyses. Descriptive statistics for age and duration of olfactory loss are summarized in Table 1.

**Table 1:** Descriptive statistics for age and duration of olfactory loss in patient and control groups.

	N	Females	Age				Duration of olfactory loss (months)			
			M	SD	Min	Max	M	SD	Min	Max
Controls	97	61	48.3	17.6	19	85				
Patients	62	33	60	13.9	29	85	33.7	46.1	1	240

### The course of the intervention

The duration of the intervention for patients and healthy controls is summarized in Table 2.

**Table 2:** Descriptive statistics for the duration of the intervention [in days] across the two groups.

Group	N	M	SD	Min	Max
Controls	97	13.59	1.50	8	18
Patients	62	13.52	1.68	5	16
Total	159	13.56	1.57	5	18

During the 14 days individual participants noted from 0 to 362 olfactory perceptions throughout each day, resulting in the total number of 55,444 olfactory perceptions. An average number of olfactory perceptions per day was *M* = 25.8 ± 30.8. The number of reported olfactory perceptions decreased with age, *r* =  − 0.31*, p* < 0.001. In the total sample, women (*M* = 30.1 ± 2.9 [24.4; 35.7]) reported smelling more odours per day than did men (*M* = 20 ± 3.4 [13.2; 26.8])*, F*(1,157.1) = 5.1, *p* = 0.03. Descriptive statistics are presented in Table 3.

**Table 3:** Descriptive statistics for the number of olfactory perceptions for both study groups split by the number of interpersonal encounters and environment.

Interpersonal encounters	Environment	Sex	Controls (n = 97)			Patients (n = 62)		
			N	M	SD	N	M	SD
Few	Indoor	Men	131	29.20	24.30	78	6.28	3.84
		Women	170	32.41	44.24	103	19.47	19.43
	Indoor and outdoor	Men	67	24.72	25.01	91	6.79	4.61
		Women	107	27.13	23.28	65	18.82	17.19
	Outdoor	Men	42	30.95	24.19	49	4.73	4.34
		Women	69	36.35	37.23	15	18.53	23.37
Many	Indoor	Men	136	33.50	26.45	47	10.28	10.20
		Women	267	44.80	44.31	126	20.52	14.79
	Indoor and outdoor	Men	55	42.13	51.60	28	10.07	10.19
		Women	114	31.51	28.42	50	35.02	26.99
	Outdoor	Men	16	33.06	20.03	19	4.63	7.10
		Women	50	33.76	23.82	11	20.73	26.64

### Correlates of the number of olfactory perceptions

The number of odours perceived daily was moderately positively correlated with baseline threshold (*rs* = 0.41, *p* < 0.001) and identification (*rs* = 0.48, *p* < 0.001) scores, individual significance of olfaction (Association: *rs* = 0.24, *p* < 0.001; Application: *rs* = 0.25, *p* < 0.001; Consequence: *rs* = 0.20, *p* < 0.001), self-rated olfactory performance (*rs* = 0.48, *p* < 0.001) and self-rated nasal patency (*rs* = 0.12, *p* < 0.001) but not depressive symptoms (*rs* = −0.03, *p* = 0.16).

### Effects of sex, environment and interpersonal encounters on the number of odors reported by patient and control groups

**Figure 1:**
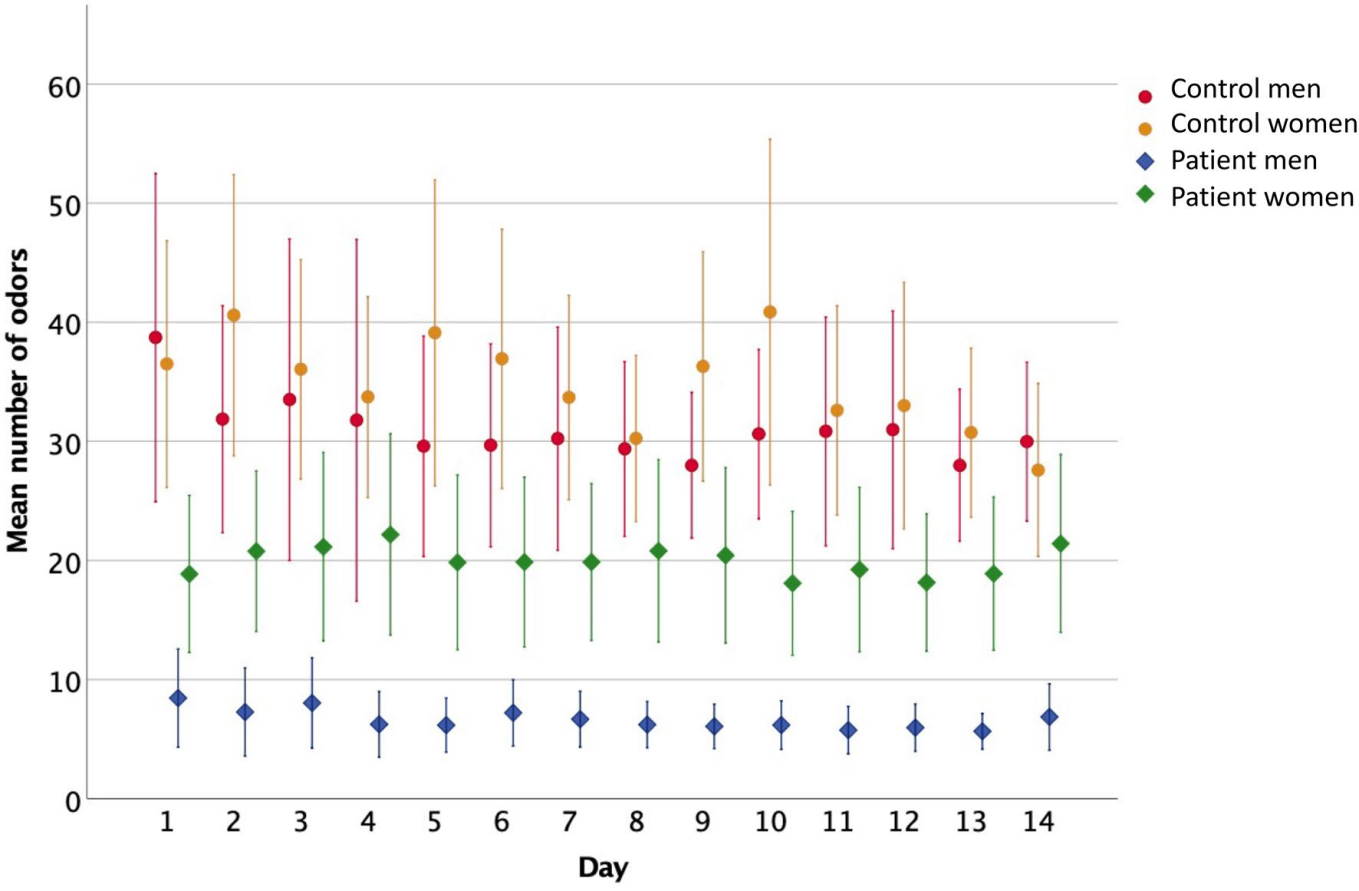
The mean number (± 95% confidence intervals) of olfactory perceptions reported by patients and controls of both sexes across the 14 days of the intervention.

The omnibus linear mixed model (oLMM) revealed a significant main effect of group on the reported number of odours, *F*(1,144) = 17.6, *p* < 0.001 with post-hoc pairwise comparisons indicating significantly more frequent odour perceptions in the control group (*M* = 35.9 ± 3 [29.9; 41.9]) than in the patient group (*M* = 14.2 ± 4.2 [5.9; 22.6]; Fig. 1). Thus, we decided to further calculate models separately for the patient and control groups.

Model examining the effect of sex, environment and interpersonal encounters on the number of odours reported by patients revealed a significant three-way interaction, *F*(2,625) = 4.23*, p* = 0.015 (all statistical coefficients for this model are reported in Table 4). Pairwise comparisons showed that across the three environments and despite the frequency of interpersonal encounters during each day of the intervention, women consistently reported more olfactory experiences than men (all *ps* < 0.004) (see Fig. 1). Additionally, women who declared frequent interpersonal contacts during the day spent in the mixed indoor-outdoor environment reported more olfactory experiences (*M* = 35 ± 2.1 [30.1; 39.1]) than those women who also met many people throughout the day but stayed mostly indoors (*M* = 20.5 ± 1.3, [17.9; 23.1], *p* < 0.001) or mostly outdoors (*M* = 20.7 ± 4.5, [11.9; 29.5], *p* = 0.012). Finally, women who spent time in the mixed indoor-outdoor environment, more frequently met other people counted more olfactory perceptions throughout the day than for women who also stayed in the mixed environment but met few people (*M* = 18.8 ± 1.8, [15.2;22.4], *p* < 0.001).

**Table 4:** Tests of fixed effects for the linear mixed models examining sex, environment, and interpersonal encounters as factors for the number of olfactory experiences reported separately for patients and controls.

	Patients				Controls			
	df1	df2	F	*p*	df1	df2	F	*p*
Intercept	1	76.40	36.97	< 0.001	1	93.9	100.5	<0 .001
Sex	1	48.83	13.81	0.001	1	93.9	0.7	0.42
Environment	2	627.27	3.10	0.046	2	1146.3	7.4	0.001
Interpersonal encounters	2	623.22	7.26	0.001	1	1140.1	15.5	< 0.001
Sex * environment	2	627.27	4.67	0.01	2	1146.3	1.4	0.25
Sex * interpersonal encounters	1	624.32	0.33	0.57	1	1140.1	0.1	0.82
Environment * interpersonal encounters	2	624.91	1.21	0.30	2	1134.4	0.0	0.96
Sex * environment * interpersonal encounters	2	624.91	4.23	0.02	2	1134.4	1.1	0.32

The model examining the effect of sex, environment and interpersonal encounters on the number of odours reported by the control group proved the frequency of interpersonal encounters to be the only significant main effect, *F*(1,1140) = 15.5, *p* < 0.001 (Table 4), wherein people declaring many interpersonal contacts during the intervention declared significantly more odour perceptions (*M* = 36.5 ± 2 [32.5; 40.5]) than those who declared meeting few people (*M* = 30.1 ± 1.6 [26.9; 33.3]). There was also a significant main effect of environment *F*(2,11,406 = 7.4, *p* = 0.001, pointing to the indoor environment (*M* = 31.7 ± 3.6 [24.6; 38.9]) as less odorous than outdoor (*M* = 39.8 ± 4.1 [31.8; 47.8], *p* = 0.002) or mixed indoor-outdoor environment (*M* = 36.2 ± 3.7 [28.8; 43.6], *p* = 0.023), but the two latter eliciting similar numbers of olfactory perceptions (*p* = 0.42).

### Effects of the intervention on olfactory performance

**Figure 2:**
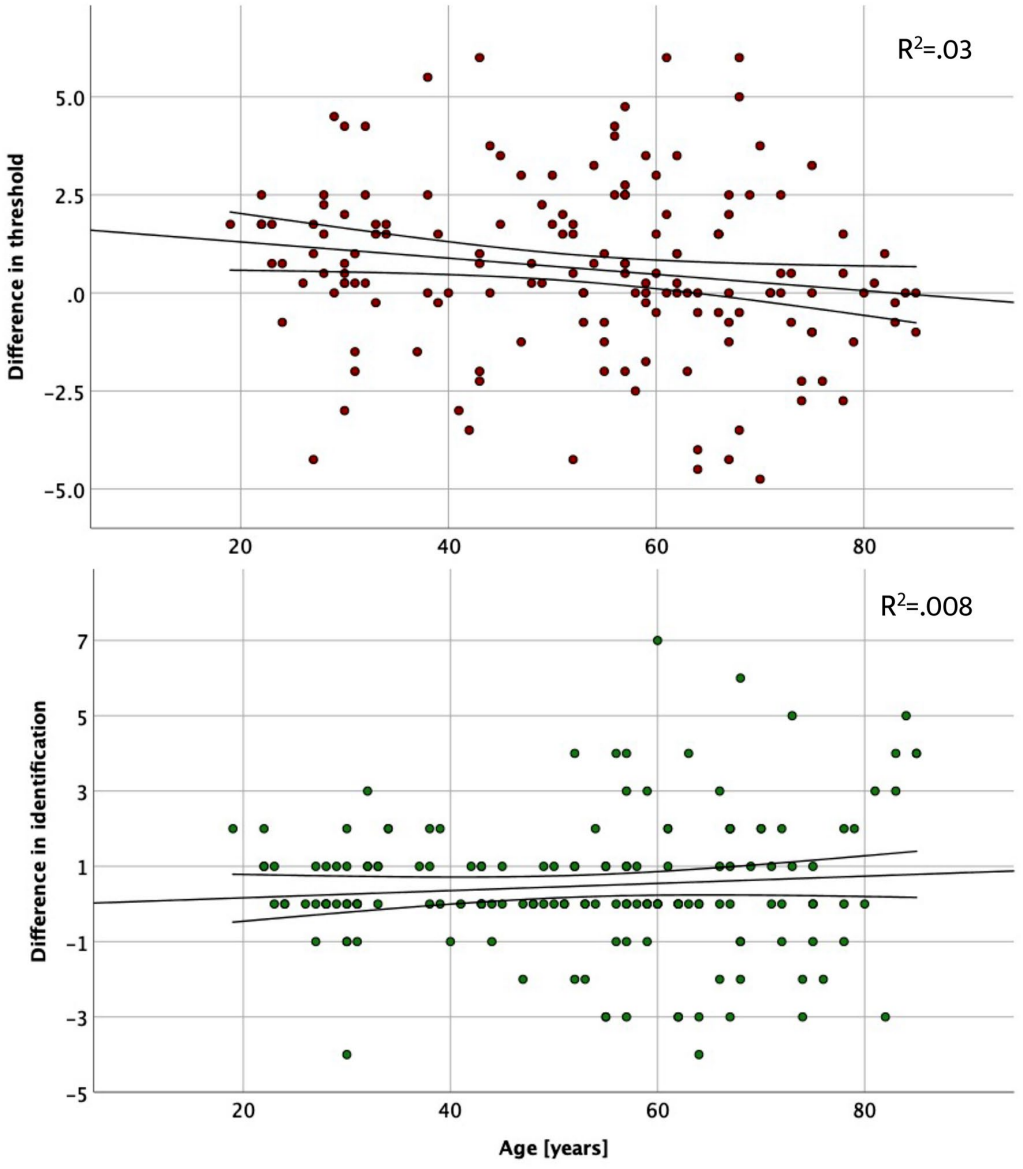
Association between the difference in olfactory sensitivity and odour identification [post- intervention measurement–pre- intervention measurement] and age. *Note*: dots above zero denote subjects who exhibited improvement in olfactory sensitivity, whereas dots below zero denote those whose olfactory sensitivity declined between the measurements.

Linear mixed model (LMM) comparing the change in olfactory threshold as a result of the intervention between the two groups, controlling for age, revealed a marginally significant main effect of the intervention, *F*(1,318) = 3.4, *p* = 0.06, and a significant and robust main effect of group, *F*(1,318) = 199.8, *p* < 0.001, but no interaction of these two factors, *F*(1,318) = 0.02, *p* = 0.88, suggesting a small increase in olfactory sensitivity in both groups as a result of the intervention. Age was a significant covariate, *F*(1,318) = 15.3, *p* < 0.001 suggesting decrease of threshold improvement with age. LMM analysing changes in odour identification yielded an identical pattern of results wherein main effect of the intervention was marginally significant, *F*(1,318) = 3.6, *p* = 0.06, the main effect of group was robust, *F*(1,318) = 259.3, *p* < 0.001, but these two factors did not interact, *F*(1,318) = 0.04, *p* = 0.84. Age was a significant covariate, *F*(1,318) = 26.6, *p* < 0.001 pointing to the marginal gain in odour identification with age. LMMs estimated marginal means for the pre- and post-intervention measurements of olfactory performance in the two groups are presented in Table 5. The relationship between age and the change in olfactory performance in presented in Fig. 2.

**Table 5:** Estimated marginal means for the threshold and identification scores for pre- and post- intervention measurements in patients and control groups, controlled for age.

	Group	Measurement	Mean	Std. error	95% Confidence interval	
					Lower bound	Upper bound
Odour threshold	Control	Pre-intervention	8.33	0.29	7.76	8.91
		Post-intervention	8.99	0.39	8.42	9.56
	Patients	Pre-intervention	3.50	0.37	2.77	4.22
		Post-intervention	4.05	0.37	3.33	4.77
Odour identification	Control	Pre-intervention	13.30	0.24	12.83	13.76
		Post-intervention	13.75	0.24	13.28	14.21
	Patients	Pre-intervention	8.40	0.31	8.12	9.30
		Post-intervention	8.89	0.30	8.68	9.85

### Personal determinants of the effectiveness of the intervention

Regression model looking into the predictive value of self-rated olfactory performance, nasal patency, depressive symptoms, individual significance of olfaction (Association, Application, and Consequence) and the average number of olfactory perceptions for the changes in olfactory sensitivity as a result of the intervention yielded no significant results. In the regression model predicting the change in odour identification, we found that higher ADS-L scores (B = 0.05, *p* = 0.008) and consequence subscale scores (B = 0.19, *p* = 0.002) predicted an increase in odour identification abilities. Regression analysis coefficients are presented in Table 6 with significant effects in bold.

**Table 6:** Self-rated olfactory performance, self-rated nasal patency, depressive symptoms, individual significance of olfaction and the average number of olfactory perceptions over the intervention period regressed on the change in olfactory sensitivity and identification as a result of the intervention.

	Difference in threshold					Difference in identification				
	B	SE	β	*t*	*p*	B	SE	β	*t*	*p*
(Constant)	− 1.62	0.96		− 1.69	0.09	− 0.25	0.78		− 0.32	0.75
Self-rated olfactory performance	0.11	0.12	0.09	0.95	0.34	− 0.04	0.10	− 0.03	− 0.36	0.72
Nasal patency	0.15	00.16	0.08	0.93	0.36	− 0.18	0.13	− 0.12	− 1.36	0.18
ADS-L	0.02	0.03	0.05	0.59	0.56	**0.05**	**0.02**	**0.22**	**2.56**	**0.01**
Association	− 0.03	0.08	− 0.05	− 0.37	0.71	− 0.10	0.06	− 0.20	− 1.66	0.10
Application	0.02	0.07	0.03	0.27	0.79	0.03	0.06	0.07	0.55	0.58
Consequence	0.14	0.08	0.20	1.77	0.08	**0.19**	**0.06**	**0.33**	**3.11**	** < 0.01**
Average number of olfactory perceptions	− 0.01	0.01	− 0.08	− 0.74	0.46	< 0.001	0.01	− 0.05	− 0.55	0.59

Significant effects were bolded.

*ADS-L*—overall depressive symptoms scale, *SE*—standard error.

### Improvement of olfactory performance as a result of the intervention

We examined the proportion of patients and control subjects in terms of clinical improvement. Subjects whose olfactory threshold increased by 2.5 points or whose identification score increased by 3 points were considered as “clinically improved”^36^. Clinical improvement of the olfactory threshold score was independent of the group χ^2^(1) = 0.04, *p* = 0.83, but clinical improvement in identification was more frequent in subjects with olfactory loss, χ^2^(1) = 5.3, *p* = 0.02. Frequencies of clinical improvement across the two groups for the difference in threshold and identification scores are summarized in Table 7.

**Table 7:** Frequencies of clinical improvement across the two groups for the difference in threshold and identification scores.

	No improvement	Clinical improvement
	Threshold	
Healthy controls	78 (80.4%)	19 (19.6%)
Patients	49 (79%)	13 (21%)
	Identification	
Healthy controls	91 (93.8%)	6 (6.2%)
Patients	51 (82.3%)	11 (17.7%)
	Threshold and identification	
Healthy controls	96 (99%)	1 (1%)
Patients	60 (96.8%)	2 (3.2%)

At the bottom rows are presented frequencies for the consistent improvement for both criteria.

## Discussion

Participants in this study reported from 0 up to 362 olfactory perceptions per day and this number highlights the frequency of olfactory stimulation and its conscious perception. The remarkable range of numbers reported by our participants shows that human olfactory ecology varies greatly between individuals. According to the “misfit” theory, people notice only those odours that stand out of the context, while odours fitting the context have a silent informative function and remain unconscious^21^. Whether an odour fits or misfits the context likely depends on individual experience with odours. Here, one of the participants reported 362 odour perceptions throughout the day making less plausible the assumption that all odours’ participants encountered throughout the day did not fit the context. Conscious perception of odours could have been enhanced by the experimenter’s request to detect odours and by wearing the finger counter, resulting in increased olfactory attention to all odours, fitting the context or not. Further, data were collected throughout the year, thus the reported effects are unlikely to be subjected to seasonal weather changes (e.g. temperature, humidity, precipitation, pollen).

Women reported perceiving odours significantly more frequently than men, but this difference was driven by the patients’ group, whereas in the control group both sexes reported similar odour counts. This study fits in the stream of consistent conclusions about the superior olfactory abilities in women^37–39^, the greater importance of the sense of smell in women's daily functioning^25,40^ and broader olfactory experience of women^20^. Our finding suggests that women with reduced sense of smell may be more motivated to pay attention to odours in their environment (or even actively seek for it), especially if they are told such practice is likely to restore their sense of smell. The lack of sex-driven difference in the control group suggests that 40 odours per day may be a ceiling effect for conscious olfactory perception. Another important demographical factor affecting the number of perceived odours was age. The number of odours smelled by the subjects decreased with age and this may reflect overall decline of olfactory performance^38,41,42^ resulting in a diminished ability to capture odorous sensations from the environment. Additionally, older people seemed less responsive to the intervention as reflected in the negative relationship between the change in odour threshold.

The richness of olfactory ecology as a function of olfactory performance is also depicted in the large difference in the number of reported odours between patients, who on an average day declared smelling 3 times fewer odours than healthy controls. Subjects reported more olfactory perceptions on the days described as filled with interpersonal contact. It implicates that those interpersonal encounters conveyed consciously perceived smells and that the interpersonal contact may constitute a source of olfactory sensations especially in people with compromised sense of smell. Thus, our results point to human chemosensory communication as an important part of olfactory ecology and that (1) the presence of other people is likely to increase alertness to odours or/and (2) odours are more easily associated with presence of other people. Odours were more frequently noted in a mixed indoor-outdoor environment, suggesting that spending a day in a diverse space likely promotes the quantity of olfactory perceptions.

The intervention we have designed to explore human olfactory ecology turned out to have limited, yet positive association with an increase in olfactory performance suggesting that (1) the conscious focus on odours may enhance olfactory performance, (2) social and physical environment can effectively stimulate the human olfactory system. However, this conclusion only holds if the perception of odours is conscious and recorded (here on a finger counter). A similar study examining the effects of meditation on conscious perception of odours showed that a few days of focused odour attention is insufficient to enhance olfactory performance^35^. Prolongation of the intervention could yield better outcomes for olfactory performance^43^ presumably due to the repeated testing and/or spontaneous recovery of the olfactory system, but it could also make the procedure cumbersome and lead to an increase of dropouts or decreased compliance. Further studies aimed to balance the length of intervention and compliance monitoring should be pursued.

Although we failed to determine factors that would significantly predict the change in olfactory sensitivity, we found depressive symptoms and the role attributed to odours in making everyday decisions as being significantly linked with the improvement of odour identification. Patients with mild to severe depression often present reduced olfactory performance^3,44^ and may not be likely to engage to a structured olfactory stimulation^45^. However, another study on olfactory training with older subjects exhibiting mild depressive symptoms reported significant improvement of olfactory function and reduction of depressive symptoms^33^. The link between depressive symptoms and human olfactory ecology should be further explored.

The present study, being a natural experiment, has certain limitations. The ability to mindfully perceive and process multiple odours in a space filled with various other sensory stimuli, may require a certain degree of attention, and focus while systematic counting requires consequence and motivation. These factors were not controlled in the current design but should be in the spotlight in the future studies aiming to unravel the psychological underpinnings of human olfactory ecology. Furthermore, odour discrimination measures could be implemented in the future. Given the nature of the intervention, ability to distinguish odours in the multi-odorant daily environment may benefit most from an intervention based on the conscious perception of odorants. The current study lays fundament for further research, but the control of attentional processes during the intervention as well as chemosensory properties of the environment would be critical to advance our knowledge in human–environment chemosensory interactions.

Capturing only orthonasal olfactory perceptions is another potential limitation of this study. Subjects were directly instructed not to record retronasal olfactory sensations, although retronasal olfaction is more strongly related to the quality of life^10^ and thus odours inhaled through this route may be more important for shaping human olfactory ecology. Comparative research on the role of ortho- and retronasal odours in human olfactory ecology are recommended. This intervention was based on self-report and its accuracy lies in the systematic documentation of the olfactory perceptions. Our subjects may have not always been able to note down the perceived olfactory sensations, but, as confirmed by the subjects, the intervention was an interesting and engaging experience, making it a promising method of support of olfactory function and odour awareness. The proposed method of OT based on a mindful perception of odours naturally occurring in the environment is a promising additional treatment method for patients with olfactory dysfunction. Further research is necessary to determine if subjects are willing to pursue this method for a longer period, comparable with the structured OT (i.e. 3–4 months). An interesting question would also be whether the structured OT performed together with the proposed intervention has a cumulative effect on olfactory system rehabilitation.

The clinical improvement in either olfactory threshold or identification was noted for approximately 20% of our subjects except for the improvement in odour identification in the control group that only 6.2% of subjects scored 3 or more points higher in the post- intervention measurement than in the pre- intervention measurement. An important aspect of these results is consistency of the clinical improvement. For the majority of our participants the clinical improvement occurred for either threshold or identification. Only in three subjects we observed a consistent improvement for both scores. A structured olfactory training (OT), recommended to patients complaining about smell loss, yields relatively more consistent improvement (5.5 points in the total Sniffin’ Sticks score, i.e. combined threshold, discrimination and identification scores [TDI])^29,30,32,46^. Hence, conscious perception of environmental odours has a fragmentary impact on olfactory performance and should rather be considered as a supportive method of olfactory system rehabilitation than a recommended treatment for patients with smell loss. Importantly, improvement of olfactory function should always be considered in the context of the baseline score. Subjects with very low scores, mostly people with anosmia or advanced hyposmia can exhibit a more robust improvement than those subjects whose score falls into the range closer to normosmia. Yet, this exciting result commands deeper examination of the outcomes of human interaction with their olfactory ecology.

## Methods

### Ethics statement

The study was conducted in accordance with the Helsinki Declaration. The project was accepted by the Ethics Committee at the Medical Faculty, Technical University of Dresden (EK 475112018) and all subjects provided written, informed consent before study inclusion.

### Participants

We determined the sample size by utilizing G*Power software^47^. Within the repeated measures design with between-within group interactions (described in detail in the Statistical approach section), to obtain the power of 0.95 with alpha level set to 0.05 to detect moderate effects of f = 0.15^48^, the projected sample size was at least 148 subjects. 163 subjects participated in the study, but 4 of them did not show up for the follow-up measurement (3 healthy subjects and 1 patient) and their baseline data were discarded. Patients were recruited at the Smell and Taste Clinic of the TU Dresden and had been classified according to the current diagnostic ORL criteria for smell disorders^49^ including anterior rhinoscopy, nasal endoscopy, olfactory testing, and MR imaging which ensures correct diagnosis assignment. The ethnologies included post-traumatic (n = 7), post-viral (n = 23), idiopathic (n = 23), sinunasal disease (n = 6) olfactory loss and other (n = 3). Healthy control subjects with normal sense of smell were recruited from the general population via local advertisements. The final sample consisted of 159 subjects of whom 62 were patients. Data were collected between June 2019 and July 2020.

### Procedure

#### Measures

Subjects were invited to the laboratory twice: for the baseline measurement and the post-intervention measurement. Measurements were performed individually. During the first session, the following questionnaires were administered: (1) standardized medical interview that includes questions about the wide range of medical conditions likely to influence olfactory performance, such as diabetes, hepatitis, nasal polyps, kidney diseases, or head trauma^50^; (2) overall depressive symptoms scale ADS-L with twenty items mapping behavioural and cognitive symptoms of depression reported on the 4-point Likert type scale ranging from 1-rarely or almost never to 4—very often, almost always. Reliability reported for healthy adults ranges from Cronbach’s α = 0.89 to α = 0.92^51^; (3) individual significance of olfaction including eighteen items in three subscales: “Association” describes emotions, memories, and evaluations that are triggered by odours, “Application” refers to the extent to which a person uses his or her sense of smell in everyday life, and “Consequence” that refers to the role of olfaction in daily decisions. The reliability reported for this questionnaire is α = 0.77^25^. Results of this scale were analysed separately for each subscale; (4) self-rated olfactory performance wherein subjects compared their olfactory performance with other people using the Likert-type scale ranging from 1—“very much worse” to 7—“very much better”; (5) self-rated nasal patency rated in reference to other people with the Likert-type scale ranging from 1—“very much worse” to 7—“very much better”.

During both sessions, olfactory function was assessed using the odour threshold and odour identification subtests from the “Sniffin’ Sticks” battery^52^. In the threshold subtest subjects were repeatedly presented with triplets of pens in 20 s intervals and had to discriminate one pen containing an odorous solution of Phenylethanol (PEA; the smell of rose) from the two blanks filled with odourless propylene glycol. A staircase paradigm was used to navigate the varying concentrations wherein two subsequent correct indications of the odorous pen resulted in a decrease in concentration and one incorrect answer increased concentration. An increase/decrease was marked as a turning point. The threshold score was the mean of the last four turning points in the staircase, with the final score ranging between 1 and 16 points.

The identification subtest consists of 16 felt-tip pens with commonly familiar odours (e.g., orange, rose, cinnamon). Participants were presented with each odour and asked to select the name of the odour from a list of four descriptors (one target and three distractors). Participants scored one point for each correct answer so scores could range from 0 to 16 points.

#### Intervention

The orthonasal perception of smells was recorded via a standard, commercially available tally counter (e.g., Feeko, USA) on each day for two weeks. Participants wore the finger counter throughout the day and pressed the button every time they perceived an odour. If the same odour was perceived multiple times throughout the day, each perception was counted separately. Subjects were instructed not to record retronasal odour perceptions, for example during meals, when chewing or eating or drinking. The sum of odours displayed on the counter was noted down at the end of the day and the counter was reset for the next morning. Additionally, subjects noted whether they spent the day mostly indoors, indoors and outdoors or mostly outdoors. For each day subjects also marked whether they encountered few or many people using a binary scale with 0 denoting few interpersonal encounters and 1 marking many interpersonal contacts throughout the day (subjects defined few/many for themselves). Although the maximum time between the two measurements exceeded 2 weeks in some subjects, only records for the first 14 days were taken into account for each subject.

### Statistical analyses

Statistical computations were performed with IBM SPSS v 25 with the level of significance set to α = 0.05. All linear mixed models had restricted maximum likelihood. We analysed sex-related differences in the number of odorous perceptions with the linear mixed model with sex included as a fixed factor and intercepts varying as a function of subjects’ ID.

To assess external validity of the number of reported odours we computed nonparametric Spearman’s *rho* correlations between the daily odour reports and baseline threshold, identification, individual significance of olfaction, self-rated olfactory performance, self-rated nasal patency and depressive symptoms. Further, we examined the number of odours reported by our subjects during the mindful odour perception intervention with an omnibus linear mixed model (oLMM) wherein we included sex (women vs men), group (patients vs controls), interpersonal encounters (few vs. many) and environment (mostly indoor vs indoor and outdoor vs. mostly outdoor) as the fixed factors and allowed intercepts to vary as the function of subjects’ IDs (random factor). Given the robust effect of group, we have further split the oLMM into two linear mixed models (LMMs) computed separately for patient and control groups. Next, to address the determinants of olfactory performance improvement between the measurements, we computed two LMMs with group (patients vs. controls) and intervention (pre- and post-intervention measurement) as fixed factors, age as a fixed covariate and intercepts varying as a function of subjects’ ID. The first model concerned odour threshold scores and the second model concerned odour identification scores. We used regression analysis to test whether self-rated olfactory performance, nasal patency, depressive symptoms and individual significance of olfaction predict outcomes of the intervention (the difference in olfactory sensitivity [post-intervention—baseline olfactory threshold score] and the difference in odour identification [post-intervention—baseline odour identification score]). We assessed the independence of clinical improvement (an increase of 2.5 points in threshold score or 3 points in identification score) from the group by the means of the χ^2^ distribution. Multiple pairwise comparisons were Bonferroni corrected.
